# Supplementary material for: Crawling and Gliding: A Computational Model for Shape-Driven Cell Migration
Source: PLoS Comput Biol. 2015 Oct 21;11(10):e1004280. doi: 10.1371/journal.pcbi.1004280 (PMC4619082; doi:10.1371/journal.pcbi.1004280)
Supplement: S1 Code — (ZIP) [file pcbi.1004280.s012.zip › release/tst/doc/html/misc_8h.html]

Tissue Simulation Toolkit: misc.h File Reference


Tissue Simulation Toolkit
 0.1.4.1


- Main Page
- Namespaces
- Classes
- Files

- File List
- File Members

Macros |
Functions

misc.h File Reference

This graph shows which files directly or indirectly include this file:

Go to the source code of this file.

|  |  |
| --- | --- |
| Macros | |
| #define | FALSE   0 |
|  | |
| #define | TRUE   1 |
|  | |
| #define | OK   1 |
|  | |
| #define | REMARK   56 |
|  | |

|  |  |
| --- | --- |
| Functions | |
| int | ReadNumber (FILE \*file, int \*number) |
|  | |
| int | YesNoP (const char \*message) |
|  | |
| char \* | GetFileName (const char \*message, const char \*ftype) |
|  | |
| int | FileExists (FILE \*\*fp, const char \*fname, const char \*ftype) |
|  | |
| int | ReadDouble (FILE \*file, double \*number) |
|  | |

## Macro Definition Documentation

#define FALSE   0

Referenced by FileExists(), FileExistsP(), GetFileName(), OpenFileAndCheckExistance(), OpenReadFile(), and YesNoP().

#define OK   1

Referenced by ReadDouble(), and ReadNumber().

#define REMARK   56

Referenced by ReadDouble(), and ReadNumber().

#define TRUE   1

Referenced by FileExists(), FileExistsP(), GetFileName(), OpenFileAndCheckExistance(), OpenWriteFile(), X11Graphics::ReplaceBeast(), and YesNoP().

## Function Documentation

|  |  |  |  |
| --- | --- | --- | --- |
| int FileExists | ( | FILE \*\* | *fp*, |
|  |  | const char \* | *fname*, |
|  |  | const char \* | *ftype* |
|  | ) |  |  |

References FALSE, and TRUE.

Referenced by GetFileName().

|  |  |  |  |
| --- | --- | --- | --- |
| char\* GetFileName | ( | const char \* | *message*, |
|  |  | const char \* | *ftype* |
|  | ) |  |  |

References FALSE, FileExists(), TRUE, and YesNoP().

|  |  |  |  |
| --- | --- | --- | --- |
| int ReadDouble | ( | FILE \* | *file*, |
|  |  | double \* | *number* |
|  | ) |  |  |

References OK, and REMARK.

Referenced by Info::Menu().

|  |  |  |  |
| --- | --- | --- | --- |
| int ReadNumber | ( | FILE \* | *file*, |
|  |  | int \* | *number* |
|  | ) |  |  |

PUBLIC

References OK, and REMARK.

|  |  |  |  |  |  |
| --- | --- | --- | --- | --- | --- |
| int YesNoP | ( | const char \* | *message* | ) |  |


---

Generated on Thu Aug 14 2014 22:04:01 for Tissue Simulation Toolkit by  

 1.8.6
